# Pfizer-BioNTech COVID-19 Vaccine (BNT162b2) Side Effects: A Systematic Review

**DOI:** 10.7759/cureus.23526

**Published:** 2022-03-26

**Authors:** Ibrahim M Dighriri, Khaled M Alhusayni, Ahmed Y Mobarki, Ibrahim S Aljerary, Khalid A Alqurashi, Fai A Aljuaid, Khalid A Alamri, Abeer A Mutwalli, Nada A Maashi, Alwaleed M Aljohani, Abdulrahman M Alqarni, Athbah E Alfaqih, Sumiya M Moazam, Muath N Almutairi, Afnan N Almutairi

**Affiliations:** 1 Department of Pharmacy, King Abdulaziz Specialist Hospital, Taif, SAU; 2 Department of Medical Laboratory, The Regional Laboratory and the Central Blood Bank, Abha, SAU; 3 Department of Pharmacy, King Faisal Hospital, Makkah, SAU; 4 Department of Pharmacy, Taif University, Taif, SAU; 5 Department of Pharmacy, King Abdulaziz Specialist Hospital Vaccination Center, Taif, SAU; 6 Department of Medicine, Jazan University, Jazan, SAU; 7 Department of Pharmacy, King Abdulaziz University, Jeddah, SAU; 8 Department of Medicine, Umm Alqura University, Makkah, SAU; 9 Department of Medicine, Aseer Central Hospital, Abha, SAU; 10 Department of Medicine, Qassim University, Qassim, SAU; 11 Department of Pharmacy, Qassim University, Qassim, SAU

**Keywords:** bnt162b2 vaccine., allergic reaction, side effects, biontech covid-19 vaccine, pfizer vaccine

## Abstract

Vaccinations prevented severe clinical complications of COVID-19. It was considered a vital component of living endemically with COVID-19. The Pfizer-BioNTech vaccine is the first mRNA-based vaccination that enhances immunity. Resulting in various adverse effects that may emerge after vaccination. This systematic review was undertaken to assess the Pfizer-BioNTech vaccine side effects by reviewing the previous studies. A total of 107 PubMed and Google Scholar publications were screened for Pfizer-BioNTech COVID-19 vaccine side effects. Fourteen articles met the study inclusion criteria. The included searching terms were a combination of “Pfizer vaccine and Side effects,” “BioNTech vaccine and side effects,” and “BNT162b2 vaccine and side effects,” as well as all synonyms. The total number of participants in the 14 studies was 10,632 participants. Average of the most frequent side effects of 14 studies were injection site pain 77.34%, fatigue 43%, muscle pain 39.67%, local swelling 33.57%, headache 33.27%, joint pain 25.75%, chills 18.34%, fever 18%, itching 9.38%, lymph nodes swelling 7.86%, nausea 7.58%, dyspnea 7.86%,and diarrhea 6.36%. The average side effects after the first dose were 79% compared with 84% after the second dose. The average occurs side effects in females at 69.8% compared with males 30.2%. Our study reveals that side effects after the Pfizer-BioNTech vaccine are common, but they are usually mild and self-limited. Local reactions like pain at the injection site are the most common. Anaphylactic shock or severe reactions are rare. We hope that our results will reassure the public that the benefits of vaccination far exceed the dangers. Also, help reduce vaccine hesitancy among individuals worried about vaccine safety and possible adverse effects.

## Introduction and background

Coronavirus 2 (SARS-CoV-2) emerged at the end of 2019 and quickly spread worldwide, causing a significant mortality and morbidity rate. As a result, SARS-CoV-2 was labeled a global pandemic by the World Health Organization in March 2020 [1,2]. SARS-CoV-2 infections can result in many clinical symptoms, ranging from asymptomatic or mild infections to severe lung and multi-organ infections resulting in death [3,4]. In addition, new SARS-CoV-2 variations have evolved due to the high transmission rates, posing a new challenge in managing the current epidemic [5-7].

Although governments and organizations all over the globe have taken many precautions to prevent the pandemic's spread, the only way to stop the threat is to find a vaccine [8]. Pfizer-BioNTech, Moderna, Sinopharm, Sinovac, Sputnik V, Janssen (Johnson & Johnson's), and AstraZeneca were among the companies that have produced COVID-19 vaccines to combat the pandemic [9,10]. These vaccines are efficient in preventing COVID-19 infection at varying levels of efficiency; however, each type of vaccination has its unique structure, pros, and cons in efficacy, immunogenicity, and safety [11-13]. The US Food and Drug Administration (FDA) approved the emergency use of the Pfizer-BioNTech COVID-19 vaccine on December 11, 2020 [14].

The Pfizer-BioNTech vaccines rely on messenger RNA technology (mRNA). The coronavirus has a spike-shaped surface feature known as an S protein [15-18]. mRNA vaccines are a new technique that has just been developed for possible application in vaccine manufacture, and several are now being tested [19]. Pfizer-BioNTech vaccine (BNT162b2), on the other hand, is regarded as the first mRNA-based vaccination for infectious illnesses to be authorized for human use. As a result of increasing immunity, several side effects may arise after vaccination. Some of the symptoms were muscle discomfort, fatigue, headache, fever, swelling, joint pain, tingling, itching, and chills [20]. However, it is still challenging to predict all the side effects of the Pfizer-BioNTech COVID-19 vaccine's mRNA technology because it's new [21].

The adverse effects profile is a critical component of the vaccination needed by medical care systems and public health [22]. However, to our knowledge, no review study has been conducted on the side effects of the Pfizer-BioNTech COVID-19 vaccine. In this regard aim, this systematic review was undertaken to discover adverse effects of vaccinated people and to offer information on the BNT162B2 vaccine's side effects.

## Review

Materials and methods

This research follows the Preferred Reporting Items for Systematic Reviews (PRISMA) checklist requirements for systematic reviews and meta-analyses [23]. We searched in PubMed and Google Scholar databases for studies related to the side effects of the Pfizer-BioNTech COVID-19 vaccine. From 2021 to 2022, to pick eligible research publications. Several medical subject headings terms were used for searching purposes, including a combination of “Pfizer vaccine and Side effects,” “BioNTech vaccine and side effects,” and “BNT162b2 vaccine and side effects” as well as all synonyms. All titles and abstracts generated from this primary investigation were rigorously edited to ensure no relevant studies were missing. Following that, the data were analyzed to identify only original research papers assessing the side effects of the Pfizer-BioNTech COVID-19 Vaccine. The second stage was to establish the inclusion criteria and exclusion criteria for studies. Abstracts were manually screened to identify relevant studies for revision. The inclusion criteria were studies on the side effects of the Pfizer-BioNTech COVID-19 Vaccine. In addition, cross-sectional or retrospective cohort or prospective cohort studies were included. Experimental studies, preclinical studies, and review articles were excluded. However, published research in a language other than English was excluded from the systematic review. The last step included collating and summarizing the pre-defined data from the final record of qualifying articles. The data evaluation process began with a preliminary examination; data were retrieved using a specifically constructed excel sheet. The excel sheet was then used to update selected data from eligible research publications. Finally, we analyzed all studies published by a single research group that examined comparable factors for any potential duplication.

Result

**Figure 1:**
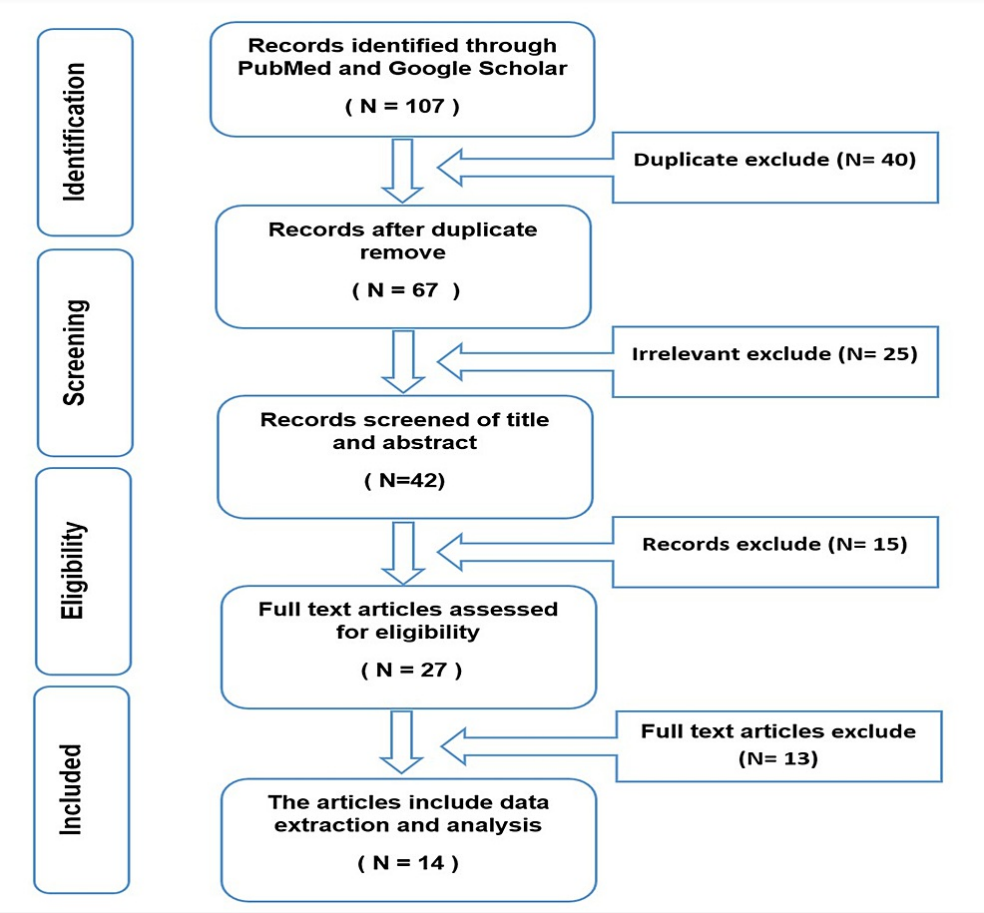
Study selection process using preferred reporting items for systematic reviews and meta-analyses (PRISMA).

A total of 107 PubMed and Google Scholar publications were screened for Pfizer-BioNTech COVID-19 vaccine side effects. Forty duplicates and 25 irrelevant were removed, and 42 articles were reviewed at the abstract level. Fifteen irrelevant abstracts were removed. After removing abstracts not meeting the inclusion criteria, 27 full-text articles were reviewed. Of these, 13 articles did not meet the inclusion criteria. The exclusion was because did not include cross-sectional or retrospective cohort or prospective cohort studies or articles in a non-English language. Fourteen articles met the study inclusion criteria and contributed to this systematic review (Figure 1).

**Figure 2:**
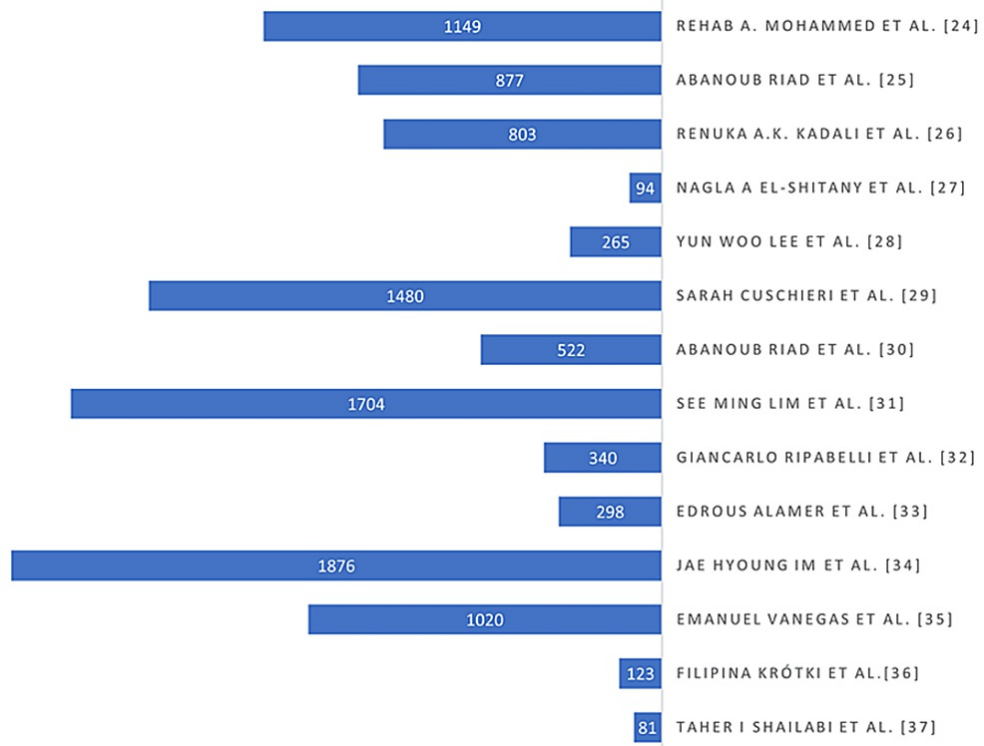
The number of participants in each study who received the Pfizer-BioNTech vaccine and reported at least one or more side effects.

The total number of participants in the 14 studies was 10,632, who reported at least one or more side effects (Figure 2). Maximum participants in Im et al.'s article were around 1,876. Minimum participants in Shailabi et al. article were only 81 participants. The mean age of all participants in 14 studies was 35.3 ± 11.1 years old. Of all participants, average males were 30.2% and females 69.8%.

All studies were published between 2021 and 2022. Of 14 studies, 13 were published in 2021 and only one in 2022. Characteristics of the selected articles are summarized in Table 1. All included studies were written in English. Side effects on the local level were more prevalent than adverse effects on the systemic level. Females suffer more side effects than males. Following the second dosage, there were greater adverse effects than the first. After the first dose, the average side effects were 79% and 84% after the second dose. However, in most subjects, adverse effects were mild and short-term. Anaphylactic shock or severe reactions are rare.

**Table 1:** The characterization of included studies in the systematic review.

Author and publication year	Method	Result and main findings
Mohammed et al. [24] 11/3/2021	A cross-sectional study involving adults of various ages and genders. Between February and March 2021, a validated questionnaire was distributed via social networking sites to Saudi Arabian residents via a Google form.	The subjects were predominantly Saudi nationals (95.3 %) and were almost gender-balanced. Subjects had to be at least 18 years old. Local pain (79.3%), fatigue (42%), muscle pain (39%), local swelling (27.7%), joint pain (23.1%), headache (21.8%), fever (21.0%), chills (15.5%), local redness (14.8%), and nausea (7.3%) were the most frequently reported symptoms, with no reports of anaphylaxis, facial paralysis, or syncope. Following the second dose, there were more adverse events than following the first (p<0.001). Female gender more significant number of side effects following both vaccine doses.
Riad et al. [25] 1/4/2021	Between January and February 2021, a cross-sectional survey of healthcare employees in the Czech Republic was undertaken. A validated questionnaire with twenty-eight multiple-choice questions was employed in the investigation.	The mean age of respondents was 42.56 ±10.5 years old. The most frequently reported adverse events were pain at the injection site (89.8%), fatigue (62.2%), headache (45.6%), muscle pain (37.1%), and chills (33.9%). Adverse events were more common in the 43-year-old group, and their duration was mostly one day (45.1%) or three days (35.8%) after the immunization. Individuals who got two doses saw a statistically significant increase in adverse events. In terms of length, 45.1 % of all adverse effects lasted less than a day. By contrast, 35.8% lasted three days, 9.4 % lasted five days, 5.3 % lasted one week and 3% lasted more than a week.
Kadali et al. [26] 15/4/2021	The side effects of the BNT162b2 vaccine were investigated in a randomized, cross-sectional study using an independent online questionnaire distributed to healthcare workers (HCWs).	Of all HCWs, 64.5% (803/1245) received the BNT162b2 vaccine and reported side effects. localized symptoms, 719/803 (89.54%); generalized symptoms, 610/803 (75.97%). musculoskeletal symptoms, 428/803 (53.3%); gastrointestinal symptoms, 172/803 (21.42%); psychological symptoms, 133/803 (16.56%); neurological symptoms, 102/803 (12.7%); head/ear/eyes/nose/throat symptoms, 97/803 (12.08%); endocrine symptoms, 83/803 (10.34%); cardiovascular symptoms, 48/803 (5.98%); respiratory symptoms, 21/803 (2.61%) and urinary symptoms, 10/803 (1.24%).
El-Shitany et al. [27] 19/4/2021	A Google Form questionnaire gathered a cross sectional study conducted retrospectively. All participants were Saudi Arabia inhabitants. Adverse effects of the vaccine were reported after the first and the second doses.	The most frequent symptoms were injection site pain 63.8 %, headache 18.1%, bone and muscle symptoms 7.4 %, fever 4.3 %, and fatigue 3.2 %. These research findings indicated a substantial increase (p < 0.001) in the proportion of participants who were experiencing side effects after getting the second dosage of the vaccination (98.4 %) compared to those who reported side effects after the first dose. Additionally, the results indicated a significant increase (p< 0.05) in the proportion of participants reporting injection site side effects following the second dose of the vaccine (80.6 % ), compared to the proportion reporting local side effects following the first dose (70.5 % ) or both doses(63.8%).
Lee et al. [28] 11/5/2021	A survey performed a prospective and mobile based on healthcare workers' self-reported adverse responses after receiving both doses of the BNT162b2 mRNA vaccination	The study received 265 (77.5 %) responses. Adverse events occurred at a greater rate after the second dosage than after the first dose (89.1 % vs. 80.1 %, P<0.006). Muscle discomfort (69.1 %), fatigue (65.7 %), headache (48.7 %), chills (44.2 %), and fever (32.1%) were the most frequently reported systemic effects. The most common local symptoms were pain (80%), itching (9.4%), redness (9.8%), and swelling (10.2 %). Females experienced more adverse reactions than males (95% vs. 78%, P<0.001).
Cuschieri et al. [29] 6/7/2021	From 29/3/21 to 9/4/21, an online survey was sent to all caregivers through work email to collect side effects related to pain, redness, and swelling at the injection site, headache, fever, and diarrhea severity after each dosage.	The commonest side-effect was pain at the injection site 49.85 %, with the majority rating it as mild (51 %) and moderate (43 %). Fatigue was mentioned by 21.79 %, with 42 % describing it as mild and 41% as moderate. Headaches were recorded by 22 %, with 51 % mild and 34 % moderate. Younger people reported greater side effects than older persons. localized side effects were reported after both vaccination doses, unlike systemic side effects primarily reported with second dosages.
Riad et al. [30] 29/8/2021	A cross-sectional study was conducted to assess the adverse effects of vaccination on healthcare professionals in Slovakia. The research used a validated self administered questionnaire to elicit medical anamneses, COVID-19-related anamnesis, and adverse events associated with the vaccination.	This research included 522 individuals, 77% of whom were female and 55.7 % of whom were between the ages of 31 and 54. The most often reported local adverse event (85.2 %) was injection site pain, followed by injection site swelling (10.2 %) and injection site redness (8.4 % ). The difference between females (87.3%) and men (78.3%) in terms of injection site pain was statistically significant (χ2 = 5.926; Sig. < 0.015). Fatigue was the most often reported systemic adverse effect (54.2 %), followed by headache (34.3 %), muscular pain (28.4 %), chills (26.4 %), and malaise (20.5% ). Females showed a greater incidence of all requested systemic adverse events in this research. The gender differences in fatigue (χ2 = 14.215; Sig.< 0.001), headache ( χ2 = 7.089; Sig. < 0.008), and joint pain ( χ2= 4.950; Sig.< 0.026) were statistically significant.
Lim et al. [31] 15/9/2021	The Occupational Health Clinic at the National University Hospital in Singapore collected data on staff adverse reactions within 30 minutes after vaccination. In addition, cross-sectional research was undertaken among vaccinated Health workers utilizing an online survey.	The three most common symptoms listened to within the 30-minute observation period at the vaccination clinic were giddiness (32.7%), rashes/itch (23.5%), and palpitation/chest discomfort (17.2%). 196 staff requiring such care after vaccination, 16 (8.2%) of them had to be transferred to ED for further evaluation and management. Adverse reactions reported from the survey: 1,704 are complete surveys. Injection site reaction (rash, redness, swelling, pain) after the first dose is 57.2% compared with 70.1% after the second dose (OR: 2.8). Feeling unwell in general (fatigue, tiredness, weakness) after the first dose is 36.7% compared with 66.1% after the second dose (OR: 6.3). Aches and pains (joint pain, muscle pain, body ache) after the first dose is 30.1% compared with 51.9% after the second dose (OR:3.7). Headache after the first dose is 18.8% compared with 41.7% after the second dose (OR:6.1). Fever or chills after the first dose is 9.3% compared with 44.7% after the second dose (OR:22.5).
Ripabelli et al. [32] 9/10/2021	This retrospective research used data obtained anonymously from five private health clinics in central Italy. The the investigation aimed to look into each adverse event after vaccination with Pfizer-BioNTech between January and March 2021.	The 340 participated in the study. The enrolled subjects were aged 49.2 ± 13.4 years, and 209 (61.5%) were females. Most adverse events following the first dose: Pain, redness, and swelling at the injection site 265 (77.9%), Fatigue 66 (19.4%), Headache 52 (15.3%), Fever 5 (1.5%), Chills 20 (5.9%) and Sleep disorders 8 (2.4%). Most adverse events following the second dose: Pain, redness, and swelling at the injection site 223 (56.6%), Fatigue 135 (39.7%), Headache 89 (28.8%), Fever 46 (13.5%) and Sleep disorders 20 (5.9%). Adverse events were reported by 279 (82%) and 281 (82.6%) individuals as induced by the first and second dose, respectively.
Alamer et al. [33] 9/11/2021	A retrospective, cross-sectional investigation to determine the adverse events experienced by children in this age range after delivering one or two doses of the Pfizer-BioNTech vaccine. Via way of an online survey that was self-administered.	The commonly reported adverse effects were pain or redness at the site of injection (90 %), tiredness (61%), fever (39%), headache (49 %), nausea or vomiting (21 %), and chest pain and shortness of breath (18 %) Statistics indicated that more female participants experienced side effects than male participants, with 52 % and 48 %, respectively. In addition, side effects were more prevalent after the second dosage. These adverse effects continued for 1–3 days in 65 % of the individuals, 3–5 days in 30 % of the research respondents. Findings demonstrated that side effects were often reported in individuals who got two doses compared to the singly dosed subjects.
Im et al. [34] 16/11/2021	Medical practitioners were examined at a university hospital immunized with the Pfizer-BioNTech COVID-19 vaccine. Seven days after each injection, the survey was administered using a diary card. For the first dosage, 75.1 % (1876/2498) responded to the questionnaire; 73.8 % (1840/2493) responded to the second dose.	No serious adverse events occurred after the first or second dose, such as anaphylaxis. The most common local adverse event was pain, which was reported in 84.9% (1592/1876) of responders after the first dose and 90.4% (1664/1840) of responders after the second dose, followed by swelling [16.5% (309/1876) and 35.5% (653/1840), respectively] and redness [14.1% and 31.9%, respectively]. Among systemic adverse events, fatigue was the most common [52.8% after the first dose and 77.0% after the second dose], followed by myalgia [49.0% and 76.1%, respectively], headache [28.7% and 59.2%, respectively], chills [16.7% and 54.0%, respectively], itching [12.0% and 22.7%, respectively], nausea or vomiting [11.4% and 22.6%, respectively], and urticaria [2.3% and 2.7%, respectively].
Vanegas et al. [35] 18/11/2021	From March to May 2021, researchers conducted observational cross-sectional research to examine probable adverse effects of the Pfizer COVID-19 vaccination among a sample of healthcare professionals in Guayaquil, Ecuador.	The sample consisted of 1291 participants, 50.4 % female, and 41.6 % male. The sample's mean age was 39.3 years (SD, 13.5). On average, 79% (N = 1020) of participants had an unpleasant reaction after the first dosage, whereas 75.1 % (N = 969) experienced an adverse response following the second dose. Local adverse effects were more prevalent than systemic adverse effects. Most adverse events reported after the first dosage to 1020 individuals were as follows: Local side reactions included pain, erythema, edema, pruritus, and axillary edema in 896 (69.4 %). Fever, malaise, myalgia, and arthralgia are examples of systemic adverse effects 557 (43.1%). Most adverse events were reported in 969 individuals after the second dose: Local adverse reactions included pain, erythema, edema, pruritus, and axillary edema in 753 (58.3 %). Fever, malaise, myalgia, and arthralgia were 666 (51.6 %) systemic side symptoms.
Filipina Krótki et al. [36] 20/12/2021	An anonymous survey was conducted. People vaccinated with two doses of the SARS-CoV-2 vaccine (BNT162b2) was qualified for the study. One hundred twenty-three people were included in the study.	The most frequently reported adverse events were pain or swelling at the application site (91.6 %) after the first dosage, compared to (73.17 %) following the second dose. Muscles and joints ache (45.53%) after the first dosage, compared to (56.10%) following the second dose. After the first dosage, (57.72 %) reported feeling weak, compared to (74.80 %) after the second dose. Shivers (19.51%) after the first dosage, compared to (37.40%) following the second dose. Fever (18.70%) with the first dosage, compared to (40.56%) following the second dose. Headache (31.71%) occurred after the first dosage, while (46.34%) occurred after the second dose. Enlargement lymph nodes were (13.82 %) with the first dosage vs. (16.26 %) following the second dose. Dyspnoea (4.88 %) occurred with both the first and second doses.
Shailabi et al . [37] 13/1/2022	A investigation was done on healthy young people of both sexes. Eighty-one individuals were got two doses of the Pfizer-BioNTech COVID-19 Vaccine. A paper questionnaire was produced; the survey questions about the side effects of taking the Vaccine. All subjects completed the questionnaire twice, once after the first dosage and again after the second dose.	Participant's average age was (21.5 ±1.7 years). The study revealed a significant difference between the number of females (96.5%) and males (85%) who suffered from the side effects of the Pfizer-BioNTech COVID-19 Vaccine (P<0.033). Male had the highest incidence of muscle pain (70%) compared to females (66.1%). (62.6 %) of females reported feeling tired, compared to (50%) of males. For headache, the results showed a significant difference between females and males, Where the percentage of females was (60.9%), compared to (35%) for males (P<0.048). Finally, there were no clear differences in the percentage of fever between females and males, as it was (49.6%) for females and (50%) for males (P > 0.999).

Average of the most frequent side effects of 14 studies were injection site pain 77.34%, fatigue 43%, muscle pain 39.67%, local swelling 33.57%, headache 33.27%, joint pain 25.75%, chills 18.34%, fever 18%, itching 9.38%, lymph nodes swelling 7.86%, nausea 7.58%, dyspnea 7.86%, and diarrhea 6.36% (Table 2). Injection site pain considers the most often occurring local adverse effect. Followed by fatigue is considered the most often occurring systemic adverse event. Maximum side effects of injection site pain reported in Krótki et al.'s article around 91.06%. Maximum side effects of fatigue reported in Lee et al.'s article around 65.70%.

**Table 2:** Most reported side effects of the Pfizer-BioNTech vaccine in 10,632 participants from 14 studies.

Author Publication year		Mohammed et al. [24] 3/2021	Riad et al. [25] 4/2021	Kadali et al. [26] 4/2021	Shitany et al. [27] 4/2021	Lee et al. [28] 5/2021	Cuschieri et al. [29] 7/2021	Riad et al. [30] 8/2021	Lim et al. [31] 9/2021	Ripabelli et al. [32] 10/2021	Alamer et al. [33] 11/2021	Im et al. [34] 11/2021	Vanegas et al. [35] 11/2021	Krótki et al. [36] 12/2021	Shailabi et al. [37] 1/2022	Average
Number of participants		1149	877	803	94	265	1480	522	1704	340	298	1876	1020	123	81	
Side effect %	Injection site pain	79.30%	89.80 %	88.04%	63.80%	80 %	49.85%	85.20%	57.20%	77.90%	90%	84.90%	68.40%	91.06%	NA	77.34%
	Fatigue	42%	62.20%	58.89%	3.20%	65.70%	21.79%	54.20%	36.70%	19.40%	61%	52.80%	6%	57.72%	56.80%	43%
	Muscle pain	39.10%	37%	45.70%	7.40%	69%	24.16%	28.40%	30%	NA	NA	NA	NA	45.53%	70.40%	39.67%
	Local swelling	27.70%	25.60%	5.48%	NA	10.20%	36.44%	10.20%	NA	77.90%	NA	16.50%	NA	91.06%	34.60%	33.57%
	Joint pain	23.10%	27.80%	16.56%	NA	30.60%	11%	17.60%	30%	NA	NA	NA	NA	45.52%	29.60%	25.75%
	Headache	21.80%	45.60%	45.48%	18.10%	48.70%	22%	34.30%	18.80%	15.30%	49%	28.70%	NA	31.71%	53%	33.27%
	Fever	21%	21.70%	22%	4.30%	32.10%	10.11%	15.30%	9.30%	1.50%	39%	1%	11.50%	18.70%	43%	18%
	Chills	15.50%	34%	36.60%	1.10%	44.20%	10.20%	26.40%	9.30%	5.90%	19%	16.70%	3.50%	19.51%	14.80%	18.34%
	Nausea	7.30%	13%	15.94%	1.10%	NA	NA	9.40%	4.30%	2.10%	NA	11.40%	2.70%	NA	8.60%	7.58%
	Itching	NA	NA	5.35%	NA	9.40%	NA	1.50%	3.50%	0.60%	NA	NA	0.60%	NA	24.70%	9.38%
	Diarrhea	NA	NA	4.61%	NA	12.50%	13.75%	NA	4.30%	2.10%	NA	7%	2.90%	NA	3.70%	6.36%
	Dyspnoea	NA	NA	1.99%	NA	NA	NA	NA	2.50%	NA	18%	NA	NA	4.88%	7.40%	6.95%
	Lymphadenopathy	2.80%	16.20%	3.36%	NA	NA	NA	7.50%	NA	2.40%	NA	NA	NA	13.82%	NA	7.86%

Two studies included 1,634 patients. Those two studies showed the onset of symptoms after taking the BNT162b2 vaccination; 134 participants (8.2%) had symptoms within less than an hour, while 1,140 (69.9%) had symptoms within less than 24 hours and 297 (18.1%) patients started experiencing symptoms after 24 hours to 48 hours. Also, about 63 (3.9%) patients developed symptoms within more than 48 hours of receiving the BNT162b2 vaccine (Table 3).

**Table 3:** Onset of the side effects after receiving of Pfizer COVID-19 vaccine among 1,634 participants.

Percentage (%)	Frequency (N)	The onset of side effects
8.2	134	<1h
69.8	1,140	<24
18.1	297	24-48
3.9	63	>48

Discussion

Preventive measures to limit SARS-CoV-2 transmission have been implemented globally since the COVID-19 pandemic began in January 2020 [38,39].

Vaccination protects against illness and spread. Although the US FDA and each country's Department of Health and Human Services have authorized various COVID-19 vaccines for usage, their side effects have not been thoroughly discussed [40].

Any vaccination is predicted to induce transitory side effects due to the activated immune response and the injection site tissue being traumatized. The adverse effects were classified as localized or systemic [41].

This systematic review shows the average of local side effects was Injection site pain 77.34%, considered the most often occurring local adverse effect, followed by local swelling 33.57%. According to research published in 2021 by Elnaem et al., pain at the injection site (61.1%) and fatigue (48.8 %) were the most frequently reported adverse effects among individuals who got Pfizer vaccination [42].

This review shows the average systematic side effects were fatigue 43% consider the most often occurring systemic adverse event, followed by muscle pain 39.67%, headache 33.27%, joint pain 25.75%, chills 18.34%, fever 18%, Itching 9.38%, lymph nodes swelling 7.86%, nausea 7.58%, dyspnea 7.86% and diarrhea 6.36%. These were prevalent and similar to previous research, like Alghamdi et al., noted that the most common adverse effects of the vaccination were fatigue, headache, and fever [43]. According to with manufacturer, fatigue and headaches were the second and third most often reported side reactions, occurring mainly after the second dosage [44].

Our findings indicate that most side effects are mild or moderate in severity and self-limited. Also, the incidence of serious adverse events such as anaphylactic shock or allergic response was not remarkable.

In this study, according to gender differences, the average side effects occur in females (69.8%) compared with males (30.2%). Males and females respond differently to vaccination. Biological differences, such as endocrine and sex hormones, play a significant role in females' high response to bacterial and viral vaccines [45]. Sex variations in pharmacokinetics and pharmacodynamics have also been observed, with females being more susceptible to adverse effects [46]. These effects have been attributed to females having a more significant body fat percentage than men, which affects the volume of distribution and clearance of medications [47].

Our findings show that after the second dosage, there were greater adverse effects than the first. The average side effects after the first dose were 79% and 84% after the second dose. When comparing the second and first doses of the vaccine, a study issued by the US Food and Drug Administration indicated that the incidence of local adverse effects was somewhat greater following the second dosage [44]. Abu-Hammad et al. demonstrated that adverse effects were more prevalent after the second dosage [48]. According to Elnaem et al., around 40% of adverse effects occurred more often with the second dosage, especially in those who got the Pfizer-BioNTech vaccination vs. those who received the Sinovac or AstraZeneca vaccine [42].

This research started symptoms after the vaccine of 134 participants (8.2%) had symptoms within less than an hour. In contrast, 1,140 (69.9%) had symptoms within less than 24 hours, and 297 (18.1%) patients started experiencing symptoms after vaccination on the second day. Also, about 63 (3.9%) patients developed symptoms within more than 48 hours of receiving the BNT162b2 vaccine and resolved after a few days by using analgesic medications. Alhazmi et al. found the onset of adverse effects, most notably during the first and second days, at the following times: 84% on the first day, 15% on the second day, and 1% on the third or later day [49].

According to The Centers for Disease Control and Prevention (CDC) recommendations, all vaccine recipients should be watched for at least 15 minutes after immunization, with adrenaline readily accessible at the vaccination site in case it is required. Rather than the active components, it is the inactive substances or excipients (such as egg protein, gelatin, formaldehyde, thimerosal, and neomycin) that induce allergic responses. CDC advises that persons with a history of allergy to polyethylene glycol (PEG), PEG derivatives, or polysorbate avoid both m RNA Vaccines against COVID-19. Excipients (which are added to vaccines to induce a higher immunological response, prevent bacterial contamination, and maintain the vaccine's effectiveness during transit and storage) are the primary cause of vaccine-associated specific IgE-mediated and rapid responses [50,51].

The limitations of this study are searched only in PubMed and Google Scholar databases. The absence of information on certain participant groups, such as pregnant women. Exclude RCT, Case study, and case series of this systematic review. Only English-language articles were entered in this study.

## Conclusions

Our data indicate that adverse reactions to the Pfizer-BioNTech COVID-19 vaccine are common, generally mild, and self-limiting. Anaphylactic shock and severe allergic responses are rare. Local adverse effects more occur than systematic. The most often occurring local adverse effect is injection site pain and local swelling. Fatigue is the most often occurring adverse systemic event, followed by muscle pain, headache, joint pain, chills, and fever. Side effects occur more often in females than in males. These effects have been attributed to females having a more significant body fat percentage than men, which affects the volume of distribution and clearance of medications. Side effects occur more often with the second dosage than the first dose. The majority of side effects begin within 24 hours after vaccination. When the advantages of vaccination are weighed against the risks of contracting a severe COVID-19 infection or developing what seems to be largely mild to moderate short-term side effects, the benefits significantly exceed the risks. We hope that our results will reassure the public that the benefits of vaccination far exceed the risks and will help reduce vaccine hesitancy among individuals worried about vaccine safety and possible adverse effects.
